# Diversity of classic and novel human astrovirus in outpatient children with acute gastroenteritis in Shanghai, China

**DOI:** 10.3389/fmicb.2023.1265843

**Published:** 2023-11-13

**Authors:** Lijuan Lu, Huaqing Zhong, Menghua Xu, Ran Jia, Pengcheng Liu, Liyun Su, Lingfeng Cao, Xunhua Zhu, Jin Xu

**Affiliations:** ^1^Department of Clinical Laboratory, National Children’s Medical Center, Children’s Hospital of Fudan University, Shanghai, China; ^2^Shanghai Institute of Infectious Disease and Biosecurity, Fudan University, Shanghai, China

**Keywords:** human astrovirus, MLB, VA, acute gastroenteritis, children, genotype

## Abstract

**Introduction:**

Human astrovirus (HAstV) is an important pathogen of acute gastroenteritis (AGE) in children. This study was aimed at investigating the diversity and epidemiology of classic and novel HAstV in outpatient children aged 0–16 years old with AGE in Shanghai.

**Methods:**

From May 2020 to December 2022, a total of 1,482 stool samples were collected from children diagnosed as AGE from the Children’s Hospital of Fudan University. HAstV was identified using pan-astrovirus consensus primers by Reverse transcription PCR.

**Results:**

During the study period, 3.3% (49/1,482) of specimens were identified as HAstV, with a detection rate of 2.5% (37/1,482) for classic HAstV and 0.8% (12/1,482) for novel HAstV. Among the 12 novel HAstV strains, 11 (91.7%) belonged to the HAstV-MLB and 1 (8.3%) was HAstV-VA. Genotyping revealed six circulating genotypes. Strain HAstV-1 was predominant in the study population with a detection rate of 1.8% (26/1,482) followed by HAstV-MLB1 (0.7%, 10/1,482) and HAstV-4 (0.6%, 9/1,482). Of note, all the HAstV-4 strains detected in this study were close to one astrovirus strain isolated from Bactrian camels with 99.0-100.0% amino acid sequences identity. In this study, HAstV was detected in all age groups with the highest detection rate of HAstV-positive specimens observed in children older than 73 months (5.7%, 12/209).

**Discussion:**

This study provided useful information and contributed to the molecular epidemiology of both classic and novel HAstV, which were simultaneously characterized and reported for the first time in Shanghai.

## Introduction

Since their initial discovery in 1975 through electron microscopy of stool samples from infants with diarrhea and vomiting, human astroviruses (HAstV) have been associated with diarrheal and neurological infections (Appleton, 1975; Madeley and B P Cosgrove, 1975; Bosch et al., 2014; Johnson et al., 2017; Vu et al., 2017). HAstV is a well-established and significant etiological agent of acute gastroenteritis (AGE), with a worldwide distribution among young children, following rotavirus, norovirus, and adenovirus (Lu et al., 2015, 2021). Although HAstV generally causes milder illness than rotavirus and norovirus, severe HAstV cases and HAstV-associated outbreaks have been reported in various age groups and among immunocompromised patients (Oishi et al., 1994; Taylor et al., 1997; Rossit et al., 2007; Finkbeiner et al., 2009c; Johnson et al., 2017; Vu et al., 2017).

HAstV is a 28 to 30 nm icosahedral, non-enveloped, single-stranded positive RNA virus that belongs to the family *Astroviridae*. The genome length varies from 6.1 to 7.9 kb and consists of three open reading frames (ORFs), with ORF1a and ORF1b encoding the non-structural protease and polymerase proteins, respectively, and ORF2 encoding the capsid proteins (Jiang et al., 1993; Fuentes et al., 2012). Currently, the *Astroviridae* family is divided into two genera: *Mamastrovirus* species, which infect mammals, including humans, and *Avastrovirus* species, which infect poultry and other birds. Based on genome diversity, *Mamastrovirus* genera associated with human disease are further divided into four divergent species: *Mamastrovirus* 1, *Mamastrovirus* 6, *Mamastrovirus* 8, and *Mamastrovirus* 9 (Cortez et al., 2017; Johnson et al., 2017). Among these, *Mamastrovirus* 1 is designated as classic HAstVs, while *Mamastrovirus* 6, *Mamastrovirus* 8, and *Mamastrovirus* 9 are defined as novel HAstV. Classic HAstV is further classified into eight genotypes (HAstV-1 to HAstV-8). In comparison to classic HAstV, novel HAstV exhibits even greater diversity. HAstV-MLB (*Mamastrovirus* 6) is divided into three genotypes (MLB1, MLB2, and MLB3), while HAstV-VA is categorized into *Mamastrovirus* 8 species, containing VA2 (also named HMO-B) and VA4, and *Mamastrovirus* 9 species containing VA1 (also named HMO-C) and VA3 (HMO-A) (Bosch et al., 2014; Meyer et al., 2015; Vu et al., 2017).

As we all know, classic HAstV has been definitively associated with AGE, accounting for 0–29.7% of cases in children worldwide (Resque et al., 2007; Kapoor et al., 2009; Ouyang et al., 2012; Vu et al., 2017; Niendorf et al., 2022). Although all eight genotypes of classic HAstV have been linked to AGE, HAstV-1 remains the predominant genotype in most parts of the world (Ouyang et al., 2012; Lu et al., 2015, 2021; Cortez et al., 2017; Johnson et al., 2017; Wei et al., 2021). Regarding novel HAstVs, both HAstV-MLB and HAstV-VA have seen an increasing presence in stool samples collected from children with gastroenteritis since 2008. However, the definitive link between these novel HAstV infections and gastroenteritis remains unestablished due to the limited number of comprehensive epidemiological studies (Finkbeiner et al., 2008a,b, 2009a,b,c; Kapoor et al., 2009). Furthermore, HAstV has recently been identified as the causative agent of unexpected central nervous system (CNS) infections in susceptible individuals, emphasizing that these viruses may bypass the gastrointestinal tract and cause other systemic diseases (Cordey et al., 2016; Vu et al., 2016, 2017; Lulla and Firth, 2020).

Previous studies have mainly focused on the epidemiology of classic HAstV in children under 5 years of age, with limited data available on novel HAstV in Shanghai (Lu et al., 2015, 2021; Wu et al., 2020). Since the definitive association between novel HAstV infections and gastroenteritis remains unclear, it is crucial to comprehensively investigate the prevalence and diversity of HAstV in children with AGE. Additionally, epidemiological and molecular data on HAstV in children with AGE may offer valuable insights for the development of HAstV vaccines and preventive therapies in the post-rotavirus vaccine era. Consequently, this study was conducted to provide an in-depth analysis of the epidemiological characteristics of HAstV in children aged ≤ 16 years with AGE in Shanghai from April 2020 to December 2022.

## Materials and methods

### Patients and samples

A total of 1,482 fecal samples were obtained from children aged up to 16 years who visited the outpatient clinic and received a diagnosis of AGE at the Children’s Hospital of Fudan University in Shanghai, spanning from April 2020 to December 2022. AGE was defined as the occurrence of three or more loose, watery stools with a pasty texture or the presence of mucous stools per day. These symptoms may be accompanied by vomiting, abdominal pain, fever, and should last for less than 2 weeks, in accordance with the criteria outlined by the World Health Organization (WHO) (Mousavi Nasab et al., 2020; Lu et al., 2021; Wang et al., 2021). All the enrolled samples were derived from residual samples obtained after routine testing and were initially stored at –20°C when collected from the outpatient examination department before undergoing detection. Specifically, liquid and solid feces were collected in quantities ranging from approximately 500 to 1,000 microliters or the size of a mung bean, respectively. The samples were transported daily from the outpatient examination department to the research laboratories in medically refrigerated transfer cases. Additionally, demographic data for all enrolled children and clinical characteristics of children infected with HAstV were retrieved from medical records. The study protocol received approval from the Ethical Review Committee of the Children’s Hospital of Fudan University.

### Nucleic acids extraction and detection of HAstV

Fecal suspensions were diluted in 0.9% saline, vortexed, and clarified through centrifugation at 10,000 rpm for 2 min to a final concentration of approximately 10% w/v. The samples underwent total nucleic acid extraction from 200 μL of clarified stool suspensions using the Viral Ex-DNA/RNA Kit (Xi’an TianLong Science and Technology Co., Ltd.) following the manufacturer’s instructions. The viral nucleic acids that were extracted were eluted in 80 μL of diethyl pyrocarbonate (DEPC) water and subsequently stored at –80°C until they were analyzed using molecular techniques.

Reverse transcription (RT) was performed using random primers with the PrimeScript™ II Reverse Transcriptase Kit [Takara, Biotechnology (Dalian) Co., Ltd., China]. The HAstV genome was detected using the PCR method with pan-astrovirus consensus primers, SF0073 forward primer (5′-ATTGGACTCGATTTGATGG-3′, nucleotide positions 3,110–3,129) and SF0076 reverse primer (5′-CTGGCTTAACCCACATTCC-3′, nucleotide positions 3,518–3,500). These primers target the highly conserved region of ORF1b encoding RdRp in both classic and novel HAstV (Finkbeiner et al., 2009a; Jiang et al., 2013; Meyer et al., 2015; Khamrin et al., 2016; Kumthip et al., 2018). PCR amplification was carried out using the following conditions: an initial denaturation step at 94°C for 2 min, followed by 35 cycles of denaturation at 94°C for 30 s, annealing at 50°C for 30 s, extension at 72°C for 30 s, and a final extension step at 72°C for 7 min. The expected size of the amplicon was 409 base pairs. To visualize the results, four microliters of each reaction product were subjected to Super GelBlue staining (Shanghai BioScience Co., Ltd., China) and analyzed using an automated gel image analysis system (Shanghai Tanon Life Science Co., Ltd., China) following electrophoretic separation on 2.0% agarose gels.

### DNA sequencing and phylogenetic analysis

Nucleotide sequencing and the construction of phylogenetic trees were employed to determine the genotypes of the HAstV strains identified in this study. All amplification products of HAstV were subjected to sequencing using first-generation sequencing technology provided by Sangon Biotech (Shanghai) Co., Ltd. The nucleotide sequences of the detected HAstV in this investigation were compared with sequences from corresponding reference virus strains accessible in the GenBank database. Multiple sequence alignments were scrutinized using MEGA 6.0, employing the ClustalW method. Subsequently, a phylogenetic tree was constructed in MEGA 6.0, utilizing the maximum likelihood approach (Kimura 2-parameter model), and subjected to 1,000 bootstrap replicates for robustness assessment.

### Statistical analysis

Statistically significant differences in detection rates and proportions of categorical variables related to HAstV infections were assessed using Fisher’s exact test, two-tailed chi-squared test, or the corrected chi-squared test in IBM SPSS Statistics software (version 20). Two-sided *p*-values less than 0.05 were regarded as indicating statistical significance.

## Results

### Prevalence, age, and seasonality distribution of HAstV infection

Between May 2020 and December 2022, a total of 1,482 fecal samples were collected from outpatient children aged up to 16 years with AGE. Among these samples, 875 (59.0%) belonged to males, and 607 (41.0%) were females. Overall, 3.3% (49/1,482) of specimens were identified as HAstV, with detection rates of 2.5% (37/1,482) for classic HAstV and 0.8% (12/1,482) for novel HAstV. Of the 12 novel HAstV cases, 11 (91.7%) were classified as HAstV-MLB, and 1 (8.3%) as HAstV-VA. Interestingly, the detection rate of HAstV in 2021 (5.2%, 38/715) was significantly higher than that in 2020 (0.9%, 2/226) and 2022 (1.7%, 9/531) (*p* = 0.00). However, there was no significant difference (*p* = 0.883) in the HAstV detection rate between males (3.4%, 30/875) and females (3.1%, 19/607).

**FIGURE 1:**
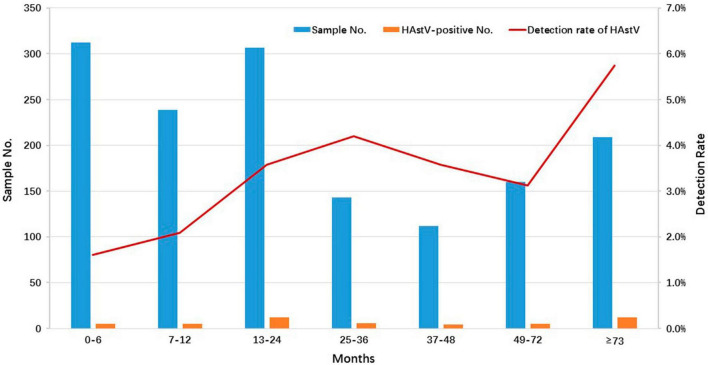
Age distribution of HAstV infections detected in outpatient children with AGE in Shanghai, 2020–2022. The number and positive rate of HAstV-infected outpatients enrolled in this study among different age groups are displayed. The blue and orange bars represent the numbers of enrolled and HAstV-positive specimens, respectively. The red line represents the positive rate of HAstV detected in outpatients with different age groups.

In this study, AGE patients were categorized into seven age groups: 0–6 months (312 cases, 21.1%), 7–12 months (239 cases, 16.1%), 13–24 months (307 cases, 20.7%), 25–36 months (143 cases, 9.6%), 37–48 months (112 cases, 7.6%), 49–72 months (160 cases, 10.8%), and over 73 months (209 cases, 14.1%) (Figure 1). Of all HAstV-positive specimens, 75.5% (37/49) of HAstV-positive children were less than 72 months of age. While HAstV was detected in all age groups, the highest detection rate of HAstV-positive specimens was observed in children older than 73 months (5.7%, 12/209), although there was no significant difference when compared to the 0–72 months age group (3.0%, 37/1,273) (*p* = 0.056) (Figure 1).

**FIGURE 2:**
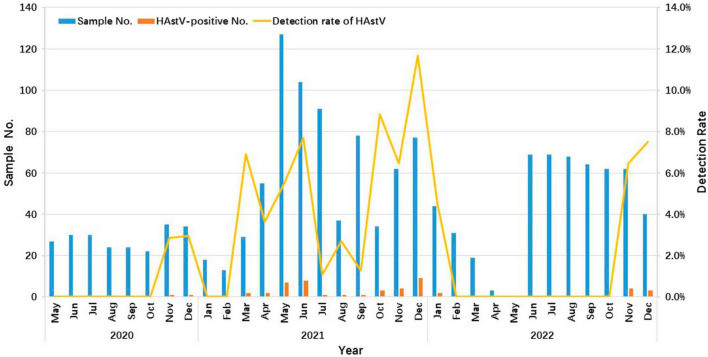
Seasonality distribution of HAstV infections in outpatient children with AGE in Shanghai from 2020 to 2022. The number and positive rate of HAstV-infected outpatients enrolled in this study from May 2020 to December 2022 are shown. The blue and orange bars represent the number of enrolled and HAstV-positive specimens, respectively. The yellow line represents the positive rate of HAstV detected in months.

The data demonstrated clear seasonal distribution patterns of HAstV over this three-year period, with a more pronounced prevalence mainly during the cold season. HAstV was detected throughout the year in 2021 but only during the winter months in 2020 and 2022 (Figure 2).

### Genotype distribution of HAstV

**FIGURE 3:**
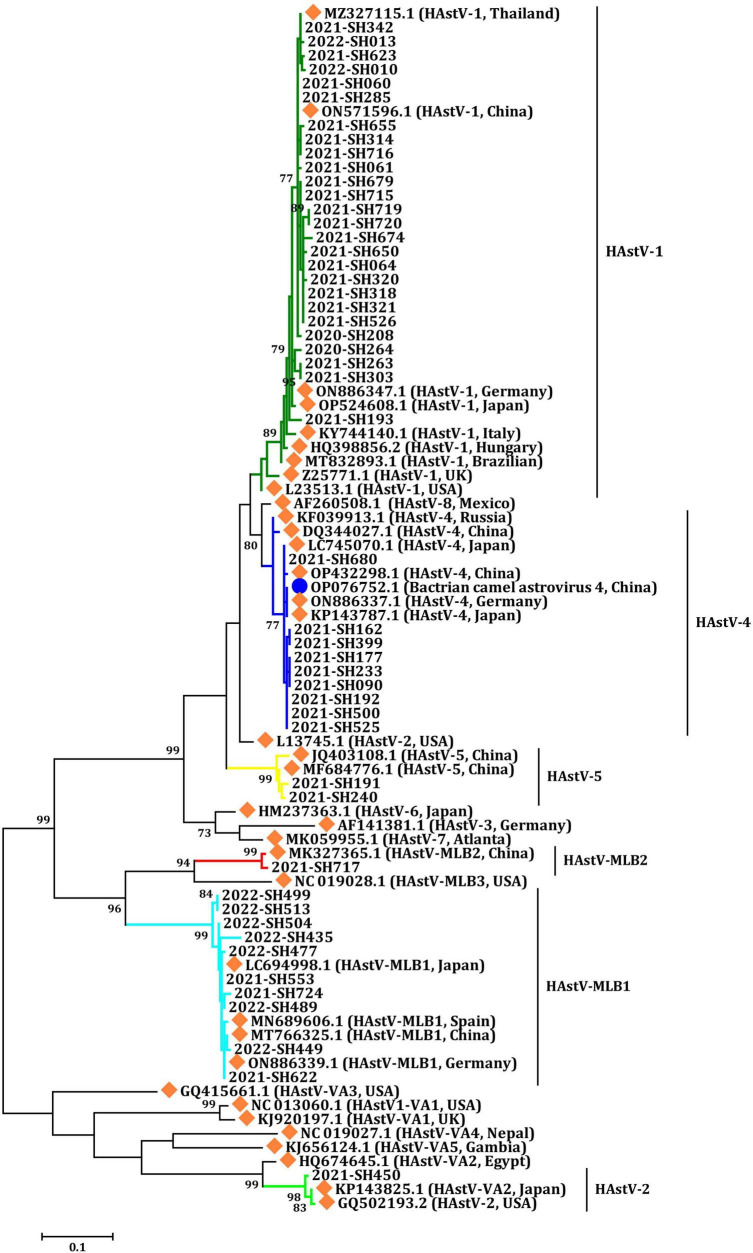
Phylogenetic analysis of HAstV according to a partial sequence of ORF1b (409 bp). Multiple sequence alignment was performed using MEGA 6.0 with the ClustalW method for the reference strains and the strains detected in this study. The trees were constructed in MEGA 6.0 through the maximum likelihood method using the Kimura 2-parameter model. The bootstrap values (1,000 replicates) are indicated in the phylogenetic tree, and values less than 70% are not represented. Orange solid diamond: HAstV reference strains. Blue solid circle: astrovirus isolated from the Bactrian camel. The others are the HAstV strains detected in this study.

In this investigation, all three primary clades of HAstV, including classic HAstV, HAstV-MLB, and HAstV-VA, were identified among outpatients with AGE. The phylogenetic analysis of partial fragments from ORF1b sequences unveiled six distinct HAstV genotypes: HAstV-1, HAstV-4, HAstV-5, HAstV-MLB1, HAstV-MLB2, and HAstV-VA2 (Figure 3 and Table 1). All six HAstV genotypes were detected in 2021, while only HAstV-1 and HAstV-MLB1 were detected in 2020, and HAstV-1, HAstV-MLB1, HAstV-4, HAstV-5, HAstV-MLB2, and HAstV-VA2 were detected in 2022.

**TABLE 1:** Demographic and clinical characteristics of children with different HAstV genotypes.

	HAstV-1	HAstV-4	HAstV-5	MLB1	MLB2	VA2	Total
	***n* (%)**						
**Gender**							
Male	18 (69.2)	3 (33.3)	2 (100.0)	6 (60.0)	–	1 (100.0)	30
Female	8 (30.8)	6 (66.7)	–	4 (40.0)	1 (100.0)	–	19
**Age**							
0–6	3 (11.5)	–	–	2 (20.0)	–	–	5
7–12	2 (7.7)	–	–	2 (20.0)	–	1 (100.0)	5
13–24	9 (34.6)	1 (22.2)	–	2 (20.0)	–	–	12
25–36	2 (7.7)	1 (11.1)	1 (50.0)	1 (10.0)	1 (100.0)		6
37–48	4 (15.4)	–	–	–	–	–	4
49–72	2 (7.7)	2 (22.2)	1 (50.0)	–	–	–	5
≥ 73	4 (15.4)	5 (55.6)	–	3 (30.0)	–	–	12
**Clinical manifestations**							
Diarrhea	26 (100.0)	9 (100.0)	2 (100.0)	10 (100.0)	1 (100.0)	1 (100.0)	49
Fever	14 (53.8)	6 (66.7)	–	3 (30.0)	1 (100.0)	1 (100.0)	25
Vomiting	8 (30.8)	4 (44.4)	2 (100.0)	3 (30.0)	1 (100.0)	1 (100.0)	16
Abdominal pain	4 (15.4)	4 (44.4)	–	3 (30.0)	–	–	11
**Patterns of clinical presentation**							
Diarrhea only	10 (38.5)	1 (11.1)	–	4 (40.0)	–	–	15
Diarrhea and fever	5 (19.2)	2 (22.2)	–	1 (10.0)	–	–	8
Diarrhea and vomiting	1 (3.8)	1 (11.1)	2 (100.0)	2 (20.0)	–	–	6
Diarrhea and abdominal pain	1 (3.8)	1 (11.1)	–	2 (20.0)	–	–	4
Diarrhea, fever and vomiting	6 (23.1)	1 (11.1)	–	–	1 (100.0)	1 (100.0)	9
Diarrhea, fever and abdominal pain	2 (7.7)	1 (11.1)	–	–	–	–	3
Diarrhea, vomiting and abdominal pain	–	–	–	1 (10.0)	–	–	1
Diarrhea, fever, vomiting and abdominal pain	1 (3.8)	2 (22.2)	–	–	–	–	3
Total	26 (100.0)	9 (100.0)	2 (100.0)	10 (100.0)	1 (100.0)	1 (100.0)	49

*n*, HAdV-positive number.

**FIGURE 4:**
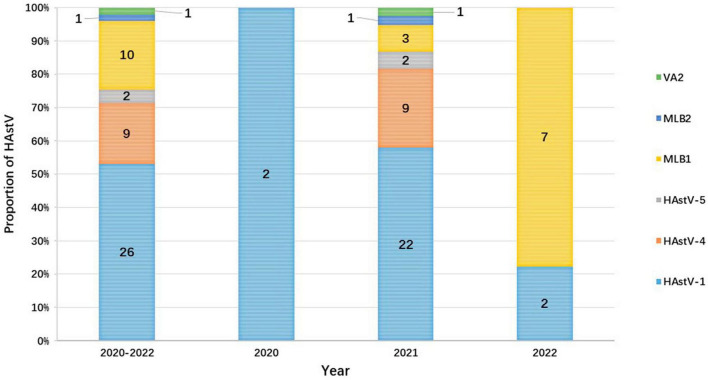
The proportion of HAstV in outpatient children with AGE each year in Shanghai from 2020 to 2022. The numbers on the bar graph represent the number of cases of each genotype detected during the year.

Human astrovirus-1 (HAstV-1) was the most common genotype with a prevalence of 1.8% (26/1,482), followed by HAstV-MLB1 with 0.7% (10 of 1,482), HAstV-4 with 0.61% (9 of 1,482), and HAstV-5 with 0.1% (2/1,482). Additionally, one HAstV-VA2 and one HAstV-MLB2 were detected in each child. However, the prevalence of HAstV genotypes varied considerably from year to year during the study. HAstV-1 was detected in all years of the study period and was the predominant genotype in both 2020 (100.0%, 2/2) and 2021 (57.9%, 22/38). However, HAstV-MBL1 (77.8%, 7/9) became the leading prevalent genotype in 2022, which was first identified in 2021. In 2021, the second most common HAstV genotype was HAstV-4 (23.7%, 9/38), followed by HAstV-MLB1 (7.9%, 3/38) and HAstV-5 (5.3%, 2/38). HAstV-1 (22.2%, 2/9) was the second predominant genotype in 2022. Furthermore, the genotypes of HAstV-4, HAstV-5, HAstV-MLB2, and HAstV-VA2 were only identified in 2021 (Figure 4).

### Gender and age distribution of HAstV genotypes

Human astrovirus (HAstV) was found in individuals across all age groups. However, the infection patterns of various HAstV genotypes exhibited distinctions concerning gender and age categories. An assessment of the gender distribution of HAstV genotypes among patients showed that HAstV-1, HAstV-4, and HAstV-MLB1 affected both males and females (Table 1). Subsequent analysis indicated that the prevalence of each HAstV genotype did not display significant variations between males and females. The patterns of HAstV genotype infections in children with age groups revealed that HAstV-1 infected children in all age groups, while the other genotypes detected in this study were dispersed in various age groups (Table 1).

### Co-infection of HAstV with other diarrheal viruses among children

All samples enrolled in this study were also detected for other common diarrheal viruses, including rotavirus (RV), human calicivirus (HuCV), and human adenovirus (HAdV). Detailed procedures were performed as previously described (Lu et al., 2015). Of the 49 HAstV-positive specimens, 14 (three novle HAstV and 11 classic HAstV) were co-infected with one or two other common diarrheal viruses. For novel HAstV, one was co-infected with RV and two were co-infected with HuCV (GI norovirus and GII norovirus, respectively). For classic HAstV, eight were co-infected with RV, two were co-infected with HuCV (sapovirus and GII norovirus, respectively), and the other one was co-infected with HuCV (GII norovirus) and RV simultaneously.

### Sequence analysis of the partial ORF1b region of HAstV

In this study, the 26 HAstV-1 and 9 HAstV-4 strains showed nucleotide sequence identities among themselves (HAstV-1: 96.2–100.0%; HAstV-4: 99.3–100%) that were higher than those when compared with the reference strains (HAstV-1: 92.0–100.0%; HAstV-4: 90.4–99.7%), respectively. The amino acid sequence identity of these 35 strains shared 96.89–100% and 95.8–100% with the reference strains used in this study, respectively. Notably, the HAstV-4 strains detected in this study were closely related to an astrovirus strain isolated from a Bactrian camel, with 99.0 to 100.0% amino acid sequence identity. Two strains of classic HAstV-5 were most closely related to reference strains reported from China, both in nucleotide sequences identity (96.5–98.6%) and amino acid sequences identity (98.9–100.0%).

The sequencing of the amplicon from only one HAstV-VA2 positive sample produced a sequence that exhibited nucleotide identities ranging from 93.0 to 98.2% with 38 reference strains worldwide available in PubMed. Additionally, the amino acid sequence of this HAstV-VA2 strain shared sequence identities ranging from 95.5 to 100.0% with 31 reference strains available in PubMed. All the HAstV-MLB1 strains collected in Shanghai displayed nucleotide sequence identities ranging from 94.8 to 100.0% with each other and shared nucleotide sequence identities ranging from 95.6 to 100.0% with reference strains from Japan, China, Spain, and Germany. However, the amino acid sequences of the 9 HAstV-MLB1 strains detected in this study were entirely consistent with the reference strains (100.0%), except for one HAstV-MLB1 strain, which shared only 94.6% amino acid sequence identity with the reference strains and the other HAstV-MLB1 strains identified in this study. The sole strain of HAstV-MLB2 detected in this study exhibited nucleotide sequence identities ranging from 93.8 to 98.8%, with 78 reference strains available in PubMed. Moreover, the amino acid sequence of this HAstV-MLB2 strain displayed sequence identities ranging from 94.6 to 100.0% with 50 reference strains available in PubMed.

### Clinical characteristics of HAstV-infected children

All children infected with HAstV exhibited diarrhea, which was one of the inclusion criteria for this study. Furthermore, among these HAstV-infected children, other common clinical features included fever (51.0%, 25/49), vomiting (38.8%, 19/49), and abdominal pain (22.5%, 11/49). The patterns of clinical presentation of HAstV-infected children varied, with 30.6% (15/49) presenting only with diarrhea, while others presented with additional common clinical symptoms besides diarrhea (Table 1).

## Discussion

Human astrovirus (HAstV) is a recognized cause of sporadic gastroenteritis, particularly in children and immunocompromised individuals. In Shanghai, previous studies have primarily focused on classic HAstV infections in children under 5 years old (Lu et al., 2015, 2021; Wu et al., 2020). The prevalence of novel HAstV infections in this region remains unclear. This study aims to comprehensively document the epidemiology of classic and novel HAstV infections in children aged 0–16 years in Shanghai.

The overall infection rate of HAstV in children with AGE in this investigation was 3.3%. This rate closely resembled findings in Thailand (2.9%, 2011–2016) but was lower than those in Japan (16.4%, 2012–2013), Kenya (11.6%, 2008), Gambia (8.3%, 2008–2009), and Brazil (7.1%, 2005–2011) (Meyer et al., 2015; Xavier Mda et al., 2015; Khamrin et al., 2016; Kumthip et al., 2018). The disparity in HAstV infection rates globally could potentially be attributed to disparities in social and geographical factors. Furthermore, HAstV has demonstrated sufficient stability in the environment to facilitate transmission through the consumption of fresh or sea water. With the enhancement of sanitation practices and public awareness of hygiene, the incidence of HAstV infections in children has evidently diminished in various regions worldwide in recent times (Abad et al., 1997; Le Guyader et al., 2000; Vu et al., 2017). Additionally, the detection rate of HAstV in 2021 (5.2%) was significantly higher compared to that in 2020 (0.9%) and 2022 (1.7%). It is hypothesized that the reduced detection rate might be linked to the implementation of non-pharmaceutical interventions (NPIs) during the Coronavirus Disease 2019 (COVID-19) pandemic, especially in 2020 and 2022 in Shanghai (Jia et al., 2022; Tan et al., 2023).

In this research, classic HAstV was the predominant HAstV, but the detection rate stood at only 2.5%, which was lower than the rate observed in our prior study from 2017 to 2018 (5.2%) (Lu et al., 2021). While classic HAstV dominated in children with AGE in most areas where all three clusters of HAstV were reported, novel HAstV has gained prominence in Japan, Germany, Kenya, and Gambia (Meyer et al., 2015; Khamrin et al., 2016; Niendorf et al., 2022). Similarly to the Thai study, the detection rate of novel HAstV in this investigation was modest, with only 0.8% of samples from children with AGE identified as novel HAstV from 2020 to 2022 (Wei et al., 2021). Intriguingly, novel HAstV emerged as the predominant HAstV in children with AGE in 2022 (77.8%, 7/9 cases). Moreover, 9 out of 12 novel HAstV infections were found as mono-infections (data not shown), raising the possibility of novel HAstV’s involvement in association with AGE. Nevertheless, the connection between novel HAstV infection and AGE remains highly debated (Holtz et al., 2011; Vu et al., 2017, 2019, 2020). A case-control study conducted in Kenya and Gambia identified novel HAstV as the most common genotype in children with AGE (Meyer et al., 2015). Some studies have discovered no disparity in the prevalence of novel HAstV between case and control groups (Holtz et al., 2011; Vu et al., 2020). Consequently, more substantial evidence is warranted to elucidate the relationship between novel HAstV infection and AGE. Given that novel HAstV has become the primary HAstV in children with AGE in 2022, and considering the limited data on novel HAstV in China, it is exceedingly imperative to continuously monitor the prevalence of novel HAstV in children with AGE in Shanghai, and even throughout China (Tao et al., 2019).

An analysis of HAstV infection prevalence in children of different genders revealed that susceptibility to HAstV infection did not differ between males and females, which aligned with our prior studies in Congo and Northwest Ethiopia (Gelaw et al., 2019; Nguekeng Tsague et al., 2020; Lu et al., 2021). In this study, the lowest infection rate of 1.6% was observed in children aged 0–6 months. Beyond this age group, the infection rate exhibited a fluctuating increase with advancing age and reached its zenith in children aged > 73 months. A similar trend was observed in Thailand from 2017 to 2020, in Korea from 2013–2017, and in Shanghai from 2015 to 2016 (Kim et al., 2019; Lu et al., 2021; Wei et al., 2021). These data suggest that children aged 0–6 months may benefit from maternal antibodies, offering protection. Subsequently, the protective effect of these external antibodies wanes as children grow older. Furthermore, older children may encounter increased opportunities for HAstV exposure and infection due to expanded outdoor activities and less hygienic behaviors. This insight into the age-specific characteristics of HAstV infection in children may serve as a reference for potential future childhood vaccination policies in Shanghai. Although the seasonality of HAstV infection is disputed and seems to fluctuate according to geographic region, HAstV infection primarily occurs during the winter season (Vu et al., 2017; Lu et al., 2021). In accordance with several other global studies, the present study revealed a typical cold season distribution of HAstV infection, with a single peak occurring in December each year during 2020 and 2022 (Xavier Mda et al., 2015; Wu et al., 2020; Huang et al., 2021; Lu et al., 2021; Luo et al., 2021). Nevertheless, in Korea and Tianjin, China, a relatively high HAstV incidence was noted during the summer months (Ouyang et al., 2012; Kim et al., 2019).

Our data highlights the diversity of circulating HAstV genotypes. In this study, most samples were characterized as HAstV-1, corroborating findings from prior surveys in various regions of China before 2021, including Shandong province, Tianjin, Guangzhou, and Jiangmen (Dai et al., 2010; Ouyang et al., 2012; Huang et al., 2021; Luo et al., 2021). Surprisingly, HAstV-MLB1 emerged as the second most prevalent genotype in our study, following its initial identification in 2021. In Thailand, HAstV-MLB1 was also identified as the second most common genotype from 2017 to 2020 (Wei et al., 2021). However, data on the prevalence of HAstV-MLB1 in China have been limited recently. In line with findings from Shanghai during 2015 and 2016, HAstV-4 stood as the third most prevalent HAstV genotype (Wu et al., 2020). Additionally, two cases of HAstV-5, one of HAstV-MLB2, and one of HAstV-VA2 were detected in this study.

Our research findings indicate that the circulating HAstV genotypes in children with AGE in Shanghai have undergone changes over time. Notably, HAstV-1 was the predominant genotype in 2020 and 2021, whereas HAstV-MLB1 became the most prevalent genotype in 2022. Furthermore, HAstV-4, HAstV-5, HAstV-MLB2, and HAstV-VA2 were identified in 2021 but disappeared in 2022. This phenomenon may be linked to the comprehensive and strict implementation of NPIs, such as mask-wearing, physical distancing, lockdowns, and school suspensions, during the high prevalence periods of COVID-19 in 2020 and 2022 in Shanghai (Jia et al., 2022; Tan et al., 2023). Conversely, the return to normalcy in daily life may have created conditions conducive to the prevalence of different HAstV genotypes among children with AGE in 2021, when the prevalence of COVID-19 was low. Therefore, continuous monitoring of prevalent HAstV genotypes in Shanghai in the future is crucial to evaluate the roles of different HAstV genotypes in children with AGE.

Analysis of the RdRp (ORF1b) nucleotide and amino acid sequences of HAstV strains detected in this study revealed higher amino acid sequence identities than nucleotide sequence identities when compared to global strains reported from various countries. These data also suggest ongoing global circulation of HAstV strains, which has implications for the slow evolutionary rate of HAstV strains in the partial sequence of ORF1b observed in this study. However, to fully understand the evolutionary pattern of HAstV, further analysis of the capsid and non-structural protein genes is necessary. When examining the sequences of HAstV-4 identified in this study, we observed high nucleotide and amino acid sequence identities when calculating pairwise distances with an astrovirus isolated from the Bactrian camel (Qureshi et al., 2023). Consistent with previous reports, our data also imply that cross-species transmission may be a common mode of transmission in HAstV infection (Cortez et al., 2017; Johnson et al., 2017; Vu et al., 2017). Given that the presence of multiple genetically distinct astroviruses within individual hosts may facilitate recombination events and the potential emergence of novel astroviruses, future research should focus on monitoring astroviruses in different host species to gain a better understanding of astrovirus ecology, evolution, and emergence.

In line with previous reports of HAstV-infected patients, diarrhea, fever, and vomiting were the most common clinical symptoms observed in this study (Glass et al., 1996; Cortez et al., 2017; Wang et al., 2022). Diarrhea was the predominant symptom in children infected with HAstV, followed by fever. Approximately 70% of these children presented with additional common clinical symptoms alongside diarrhea, while only 30.6% of children exhibited diarrhea as the sole symptom.

Our study has several limitations. Firstly, although patients receiving medical care at the Children’s Hospital of Fudan University came from various parts of Shanghai, our results may not fully represent the comprehensive and accurate epidemiological characteristics of HAstV in Shanghai, as there are several other children’s hospitals in the city. Secondly, during April and May 2022, Shanghai implemented strict lockdown measures due to COVID-19, resulting in a limited number of patients with acute diarrhea visiting clinics, and few or no samples were collected during these 2 months. Finally, we only analyzed the region of ORF1b in RdRp, which means that some recombinants within the ORF1a, ORF1b, and ORF2 regions of HAstV could not be identified in this study. This will be an important focus in our future research.

In conclusion, this study marks the first investigation into the prevalence and genetic diversity of classic and novel HAstV in children aged 0–16 years in Shanghai, China. Our data offer valuable and comprehensive insights into the molecular epidemiology of HAstV infection and emphasize the presence of diverse genotypes of HAstV concurrently circulating in Shanghai, including classic and novel HAstV. These findings underscore the importance of ongoing monitoring of HAstV epidemiology in children with AGE in Shanghai.

## Data availability statement

The datasets presented in this study can be found in online repositories. The names of the repository/repositories and accession number(s) can be found below: NCBI–OR355768-OR355816.

## Ethics statement

The studies involving humans were approved by the Ethics Committee of the Children’s Hospital of Fudan University. The studies were conducted in accordance with the local legislation and institutional requirements. The human samples used in this study were primarily isolated as part of a previous study for which ethical approval was obtained. Written informed consent for participation was not required from the participants or the participants’ legal guardians/next of kin in accordance with the national legislation and institutional requirements.

## Author contributions

LL: Data curation, Investigation, Methodology, Resources, Software, Supervision, Visualization, Writing–original draft, Writing–review and editing. HZ: Data curation, Investigation, Methodology, Writing–review and editing. MX: Methodology, Resources, Software, Writing–review and editing. RJ: Investigation, Methodology, Resources, Software, Writing–review and editing. PL: Methodology, Resources, Writing–review and editing. LS: Investigation, Resources, Writing–review and editing. LC: Data curation, Resources, Writing–review and editing. XZ: Methodology, Resources, Writing–review and editing. JX: Funding acquisition, Project administration, Resources, Supervision, Validation, Writing–review and editing.
